# Ceftriaxone for Methicillin-Susceptible Staphylococcus aureus Bacteremia: A Descriptive Case Series of Nine Patients During a Cefazolin Shortage

**DOI:** 10.7759/cureus.93977

**Published:** 2025-10-06

**Authors:** Tomohide Okinaka, Hidenobu Koga, Takashi Matono

**Affiliations:** 1 Department of Infectious Diseases, Aso Iizuka Hospital, Fukuoka, JPN; 2 Department of Clinical Research Support Office, Aso Iizuka Hospital, Fukuoka, JPN; 3 Division of Infectious Disease and Hospital Epidemiology, Saga University Hospital, Saga, JPN

**Keywords:** case-series, catheter-related bloodstream infection (crbsi), cefazolin, iv ceftriaxone, methicillin-susceptible staphylococcus aureus bacteremia

## Abstract

Introduction

Cefazolin is the standard treatment for methicillin-susceptible Staphylococcus aureus (MSSA) bacteremia. During drug shortages, alternative agents such as ceftriaxone may be considered, though evidence supporting its efficacy is conflicting. We aim to describe the clinical characteristics and outcomes of patients with MSSA bacteremia treated with ceftriaxone during a nationwide cefazolin shortage in Japan.

Methods

We conducted a retrospective case series at a single tertiary care hospital. We reviewed the records of nine adult patients hospitalized between April 2019 and March 2020 who received ceftriaxone for at least half of their intravenous therapy duration for MSSA bacteremia.

Results

The median age of the nine patients was 69 years. The infection was nosocomial in seven (78%) patients. The most common sources of infection were catheter-related. All patients achieved clinical resolution. The median duration of total intravenous antibiotic therapy was 31 days. No deaths occurred within 30 days, and no microbiological relapses were observed during a 90-day follow-up period.

Conclusion

In this small case series, ceftriaxone was associated with favorable outcomes in nine patients with MSSA bacteremia, the majority of whom had non-endocarditis, catheter-related infections. While these observations are encouraging, cefazolin remains the preferred agent. The role of ceftriaxone should be further evaluated in larger studies.

## Introduction

*Staphylococcus aureus* is a common human pathogen that causes a wide range of infections, including skin and soft tissue infections, abscesses, and life-threatening bacteremia. The case fatality rate of *S. aureus* bacteremia can be as high as 23.4% [1]. For methicillin-susceptible *S. aureus* (MSSA) infections, anti-staphylococcal penicillins are considered the first-line treatment globally, with cefazolin being a well-established alternative [2]. A large retrospective study reported lower mortality rates in patients treated with cefazolin compared to nafcillin or oxacillin [3]. In Japan, where anti-staphylococcal penicillins (e.g., nafcillin or oxacillin) are unavailable, cefazolin is the standard first-line agent for MSSA bacteremia.

Ceftriaxone offers practical advantages over cefazolin, such as a once-daily dosing schedule that facilitates outpatient parenteral antimicrobial therapy (OPAT). However, evidence regarding its effectiveness for MSSA bacteremia is limited and conflicting; while some retrospective studies suggest comparable outcomes, others report higher rates of treatment failure or relapse compared to standard therapies [4-9]. In 2019, a major generic drug manufacturer in Japan ceased its supply of cefazolin due to contamination issues, leading to a nationwide shortage that lasted for approximately one year [10]. Consequently, our hospital had to use cefazolin sparingly and employ ceftriaxone as an alternative in some cases. This situation provided a unique opportunity to observe the outcomes of patients treated with ceftriaxone as an alternative agent. Therefore, the objective of this study was to describe the clinical characteristics, treatment details, and outcomes of nine patients with MSSA bacteremia who received once-daily ceftriaxone during this shortage, to explore its feasibility and safety in a real-world setting.

## Materials and methods

This retrospective case series was conducted at Aso Iizuka Hospital, a tertiary care teaching hospital in Japan. We reviewed the electronic medical records of all adult patients hospitalized with MSSA bacteremia between April 1, 2019, and March 31, 2020, to identify those treated primarily with ceftriaxone. Patients were included if they received ceftriaxone for at least half of their total intravenous antibiotic therapy duration [11]. The ceftriaxone dosage was 2 g intravenously once daily for eight patients and 1 g once daily for one patient. Patients were also excluded if they had a central nervous system infection, died within 48 hours of antibiotic initiation, or had febrile neutropenia.

Of 45 patients with MSSA bacteremia during the study period, nine who were treated with ceftriaxone met the inclusion criteria for this case series. Data extracted included demographic information, comorbidities (Charlson Comorbidity Index (CCI)), severity of illness (Pitt Bacteremia Score), source of infection, treatment duration, and clinical outcomes. Minimum Inhibitory Concentrations were determined by the broth microdilution method according to the Clinical and Laboratory Standards Institute (CLSI) guidelines.

Catheter-related bloodstream infection (CRBSI) was defined according to the clinical practice guidelines from the Infectious Diseases Society of America [12]. Uncomplicated bacteremia was defined as cases with no evidence of endocarditis, no indwelling prosthetic devices, resolution of fever within 72 hours of therapy, negative follow-up blood cultures within 48-96 hours, and no evidence of metastatic infection [12]. Clinical cure was defined as the resolution of signs and symptoms of bacteremia at the end of therapy. Microbiological relapse was assessed during a post-treatment follow-up period of 90 days.

This study was approved by the Institutional Review Board of Aso Iizuka Hospital (approval number R22040). The requirement for informed consent was waived due to the retrospective nature of the analysis.

## Results

**Figure 1:**
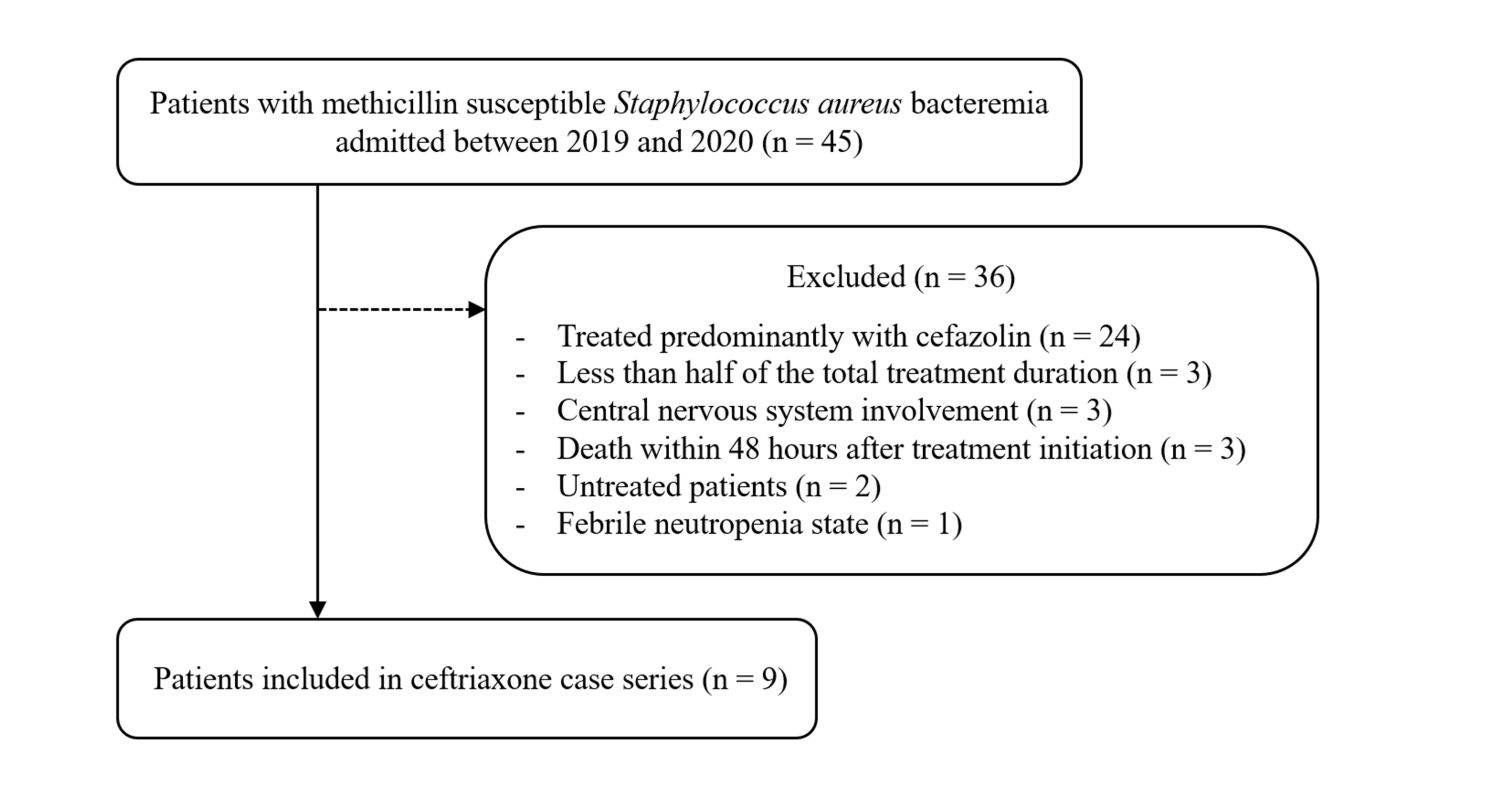
Flow diagram of patient selection From an initial cohort of 45 patients with methicillin-susceptible *Staphylococcus aureus* bacteremia, a total of 36 patients were excluded. Reasons for exclusion included treatment predominantly with cefazolin (n = 24) and other criteria such as central nervous system involvement or death within 48 hours (n = 12). The remaining nine patients were included in this descriptive case series.

Of the 45 patients with *S. aureus* bacteremia identified, nine treated with ceftriaxone met the inclusion criteria (Figure 1).

The detailed clinical characteristics, treatments, and outcomes are summarized in Table 1.

**Table 1:** Clinical characteristics and outcomes of nine patients with methicillin-susceptible Staphylococcus aureus (MSSA) bacteremia treated with ceftriaxone ABPC/SBT, ampicillin/sulbactam; CCI, Charlson Comorbidity Index; CEZ, cefazolin; CRBSI, catheter-related bloodstream infection; CTRX, ceftriaxone; IV, intravenous; MEPM, meropenem; MIC, minimum inhibitory concentration; PBS, Pitt Bacteremia Score; PIPC/TAZ, piperacillin/tazobactam; VCM, vancomycin

ID	Age	Sex	CCI	PBS	Immunosuppression	Nosocomial Infection	Source of Infection	Source Control	Antibiotics	CTRX Dosing	MIC of CTRX (μg/mL)	MIC of CEZ (μg/mL)	Time to Negative Blood Culture (Hours)	Time to Defervescence (Days)	Adverse Events	Total IV Therapy (Days)	Outcome (90-Day Survival, No Relapse)
1	69	Female	1	1	Yes	Yes	CRBSI	Catheter removed (within 24 hours)	MEPM＋VCM (six days) → CTRX (12 days)	2 g once daily	≤1	≤2	48	5	None	18	Survived
2	88	Female	2	2	No	Yes	CRBSI, suppurative thrombophlebitis	Catheter removed (within 24 hours)	ABPC/SBT＋VCM (one day) → CEZ (eight days), CTRX (23 days)	2 g once daily	≤1	≤2	24	5	None	32	Survived
3	66	Male	5	2	No	Yes	CRBSI, suppurative thrombophlebitis	Catheter removed (within 24 hours)	VCM (two days) → CTRX (20 days)	2 g once daily	≤1	≤2	24	2	None	22	Survived
4	71	Male	1	2	No	No	Cellulitis, abscess	Drainage (within 24 hours)	VCM (one day) → CEZ (four days) → CTRX (25 days)	2 g once daily	≤1	≤2	24	1	None	43	Survived
5	71	Male	1	1	No	Yes	CRBSI	Catheter removed (within 24 hours)	PIPC/TAZ (five days) → CTRX (nine days)	2 g once daily	≤1	≤2	48	2	None	14	Survived
6	69	Male	0	9	No	No	Primary bacteremia	N/A	CTRX (14 days)	2 g once daily	≤1	<=2	24	2	None	14	Survived
7	58	Female	2	1	No	Yes	CRBSI, abscess	Catheter removed and drainage (within 24 hours)	CTRX (four days) → CEZ (six days) → CTRX (eight days)	2 g once daily	≤1	≤2	48	2	None	18	Survived
8	66	Male	2	1	No	Yes	CRBSI, suppurative thrombophlebitis	Catheter removed (within 24 hours)	VCM (two days) → CEZ (six days) → CTRX (23 days)	1 g once daily	≤1	≤2	72	3	None	31	Survived
9	89	Female	1	1	No	Yes	CRBSI, suppurative thrombophlebitis	Catheter removed (within 24 hours)	VCM (two days) → CTRX (25 days)	2 g once daily	≤1	≤2	24	3	None	27	Survived

The median age was 69 years, and the median Pitt Bacteremia Score was 1, indicating a generally low severity of illness. The most common sources were related to intravascular catheters. Source control, such as catheter removal or abscess drainage, was achieved in all eight applicable cases. Ceftriaxone was used as sequential therapy in most cases; initial empirical therapy included agents such as vancomycin, piperacillin/tazobactam, or carbapenems.

All nine patients (100%) had negative blood cultures within 72 hours of treatment initiation, with a median time to negative culture of 24 hours. The median time to defervescence was two days (IQR, two to four days). No significant adverse events related to ceftriaxone were documented. All patients achieved clinical cure, and no microbiological relapses occurred during the 90-day follow-up period.

## Discussion

In this descriptive case series, we report favorable outcomes in nine patients with MSSA bacteremia who were treated with a predominantly once-daily ceftriaxone regimen, often as a sequential therapy, during a national cefazolin shortage. The main finding is that all patients achieved clinical cure and no microbiological relapses occurred during a 90-day follow-up period, in a setting where a first-line agent was scarce.

Ceftriaxone is not a first-line agent for MSSA bacteremia, largely due to concerns about its lower affinity for staphylococcal penicillin-binding proteins and a relative lack of robust clinical data. In fact, recent large retrospective studies have associated ceftriaxone with higher rates of treatment failure or mortality compared to standard therapies like cefazolin [6,9].

Our findings do not refute these larger analyses but rather provide valuable, real-world descriptive data from a unique clinical scenario where treatment options were limited. The favorable outcomes observed in our series, which contrast with findings from some larger observational studies, may be attributable to our specific patient population and management. Firstly, the cohort represented a generally low-risk group with a low severity of illness, as indicated by a median Pitt Bacteremia Score of 1. Secondly, and perhaps most importantly, prompt and effective source control was uniformly achieved in all applicable cases, such as catheter removal or abscess drainage. The success of any antibiotic regimen in bacteremia is often highly dependent on adequate source control. Thirdly, ceftriaxone was often used as sequential or de-escalation therapy after patients had been clinically stabilized on broader-spectrum empirical antibiotics. This indicates that our cohort was a selected group of patients who had already demonstrated a positive response to initial treatment.

The once-daily dosing used in our cohort highlights the potential advantage of ceftriaxone for facilitating OPAT. However, the optimal dosage of ceftriaxone for MSSA bacteremia is a subject of ongoing debate. While our patients responded well to a once-daily regimen (2 g for eight patients and 1 g for one), recent CLSI documents suggest a 2 g every 12 hours regimen for serious infections. The clinical data to definitively support either regimen remain insufficient, and this represents a key limitation of our observations and an area for future investigation.

This study has several other important limitations. First, as a small case series of nine patients, the findings are exploratory and cannot establish efficacy. Second, the absence of a control group prevents any comparative conclusions. Third, a significant selection bias likely exists, as physicians may have reserved ceftriaxone for patients perceived to have less severe illness, evidenced by the absence of endocarditis in our cohort.

## Conclusions

In conclusion, while cefazolin remains the standard of care, this series suggests that a once-daily 2 g ceftriaxone regimen may be a feasible and safe alternative for a carefully selected subgroup of patients with MSSA bacteremia. This subgroup includes patients with a low severity of illness and non-endocarditis infection sources, such as CRBSI, where prompt and effective source control is achieved. Further prospective studies are needed to clarify this role.
